# Application of artificial neural network and dynamic adsorption models to predict humic substances extraction from municipal solid waste leachate

**DOI:** 10.1038/s41598-023-39373-2

**Published:** 2023-08-01

**Authors:** Salimeh Rezaeinia, Ali Asghar Ebrahimi, Arash Dalvand, Mohammad Hassan Ehrampoush, Hossien Fallahzadeh, Mehdi Mokhtari

**Affiliations:** 1 Environmental Science and Technology Research Center, Department of Environmental Health Engineering, School of Public Health, Shahid Sadoughi University of Medical Sciences, Yazd, Iran; 2 Department of Biostatistics and Epidemiology, Research Center of Prevention and Epidemiology of Non‑Communicable Disease, Shahid Sadoughi University of Medical Sciences, Yazd, Iran

**Keywords:** Environmental sciences, Chemistry, Mathematics and computing

## Abstract

Sustainable municipal solid waste leachate (MSWL) management requires a paradigm shift from removing contaminants to effectively recovering resources and decreasing contaminants simultaneously. In this study, two types of humic substances, fulvic acid (FA) and humic acid (HA) were extracted from MSWL. HA was extracted using HCl and NaOH solution, followed by FA using a column bed under diversified operations such as flow rate, input concentration, and bed height. Also, this work aims to evaluate efficiency of Artificial Neural Network (ANN) and Dynamic adsorption models in predicting FA. With the flow rate of 0.3 mL/min, bed height of 15.5 cm, and input concentration of 4.27 g/mL, the maximum capacity of FA was obtained at 23.03 mg/g. FTIR analysis in HA and FA revealed several oxygen-containing functional groups including carboxylic, phenolic, aliphatic, and ketone. The high correlation coefficient value (R^2^) and a lower mean squared error value (MSE) were obtained using the ANN, indicating the superior ability of ANN to predict adsorption capacity compared to traditional modeling.

## Introduction

Leachate produced in the municipal solid waste landfill (MSWL) is a by-product of decomposing biodegradable waste^1^. Each year, Municipal solid waste (MSW) landfills produce hundreds of thousands of cubic meters of leachates with a high organic content^2^. Currently, the movement toward a circular economy is focused on reusing materials formerly regarded as waste into valuable resources^3^.

The successful recovery of value-added products from MSWL requires the utilization of efficient technologies^4^. Conventional MSWL treatment is frequently complicated, resulting in adverse environmental consequences and enforcing costs^5^. MSWL can be a potential resource for recovering highly value-added products^6,7^. Among the numerous substances that can be efficiently recovered from MSWL, humic substance (HS) is the most significant due to its multidirectional actions and extensive applications^8–10^.

HS is a combination of polymeric, aromatic, and aliphatic acids produced by microbial decomposition of animal and plant waste. According to HS solubility in water at various pH values, they can be operationally divided into three parts: fulvic acid (FA), humic acid (HA), and humins (Hu)^11–13^. Due to their structure and abilities, HS improve soil conditioning, root development, nutrient uptake, and plant growth^14^. Recently, these substances have been recognized as treating organic pollutants (antibiotics, herbicides, fungicides, and other phenolic compounds) and heavy metals^15–17^. Therefore, finding ways to extract HS with high efficiency is necessary. Numerous extraction techniques for HS have been developed, including nonionic or ionic resin adsorption, membrane filtering, etc^18,19^. Resin adsorption techniques are one of the most popular approaches for FA extraction because of their simplicity, easy design, affordability, and low energy consumption.

FA can be extracted by resin adsorption, recommended by the International Humic Substances Society, into different chemical groups based on its hydrophobicity. The most commonly utilized resin is Suplite DAX-8 (previously known as XAD-8). Several studies have employed DAX-8 resin to extract FA from MSWL. Baccot et al.^20^ Extracted HS from MSWL as organic amendments to improve soil structure. Similar procedures were also employed to extract HA, FA, and other organic materials using DAX-8 columns^21^.

Dynamic adsorption experiments can predict and model break-through curves (BTC_s_). They can also be easily scaled up for use in industry, making them a bridge between lab-scale experiments and real-world applications. In addition, since they can manage many solutions, they are more accurate for identifying design parameters in practical uses^22,23^. Several traditional mathematical models emerged for fixed-bed BTC_s_, including Thomas, Yoon-Nelson, and Bed Depth Service Time (BDST) models^24^. These models predict realistic adsorption efficiency without needing an experimental setup. Additionally, they give practical design information for columns^25^. However, there is a lack of information on the application of these models in the extraction of FA from MSWL using a fixed-bed.

In addition to the traditional models mentioned, the artificial neural network (ANN) model has been used to analyze the BTC_s_. ANN, a computer intelligence model influenced by biological and neurological processes, is increasingly popular for managing nonlinear processes^26^. ANN is widely recognized as a robust statistical tool due to a multitude of advantages. These advantages include the capacity to identify patterns through small modifications, the ability to approximate nonlinear systems without prior knowledge of variable relationships, simple use, and the capability to operate separately from conventional experimental designs^27^. The selection of ANN modeling was based on the features above to forecast and enhance the extraction of FS from MSWL. In recent years, its usage in the simulation adsorption processes has attracted wide attention^28^. However, little literature is reported on applying the ANN model in extracting FA with the fixed-bed column to our best knowledge.

The primary objective of this study was to establish extraction methods for obtaining HA and FA from MSWL. HA was initially isolated by applying the sample to HCl and NaOH chemicals. Subsequently, the FA was extracted utilizing a fixed-bed adsorption column under diversified operational variables such as flow rate, bed height, and input concentration. The experimental data were evaluated using the traditional Thomas, Yoon-Nelson, and BDST models and the ANN model. The models utilized to simulate and predict the extraction of FA using a fixed-bed column are novel aspects of this study. The results of this work could contribute to future efforts to extract HS from MSWL.

## Material and methods

### Landfill leachate sampling and characterization

#### Characterization Landfill leachate and HS

The leachate samples were obtained from the MSW landfill site in Yazd city, Iran. The leachate samples were carried out in polyethylene containers directly from the landfill to the lab. The samples were stored at 4 °C immediately after arrival to decrease microbial activity. Characterizations of leachate samples are presented in Table 1.

**Table 1:** Characterization of MSWL used in this study.

Parameter	Unit	Sample
pH	–	5.6
Conductivity	mS/cm	9.03
SUVA	L/mg m	2.98
DOC	mg/L	32,000
COD	mg/L	72,000
BOD	mg/L	19,000

#### Fourier transform infrared (FTIR) spectroscopy

A pellet was formed by mechanically pressing a mixture of one milligram of freeze-dried HA and FA with 100 mg of potassium bromide (KBr). The pellets were utilized for the purpose of analyzing the structure of HA and FA. FTIR spectra were obtained within the wavenumber range of 4000–400 cm^−1^ with a resolution of 4 cm and 16 scans per acquisition^29^.

#### Extraction protocol

The MSWL sample was filtered with 0.45 μm filter paper. After filtration, the sample was adjusted to pH below 2 with concentrated HCl and then refrigerated for 24 h to precipitate HA. After 24 h, solutions of 0.1 N NaOH were used to dissolve HA filtered through 0.45 μm filter paper. Following HA isolation, only FA and the hydrophilic fraction remained. The FA in the MSWL was extracted using a fixed-bed column, and the remaining stream was a nutrient-rich solution^30,31^. Following adsorption, the resins enriched with FA must be subsequently regenerated by 0.1 M NaOH, and the performance of resin reusability was assessed in this work^32^.

#### Column experiments

**Figure 1:**
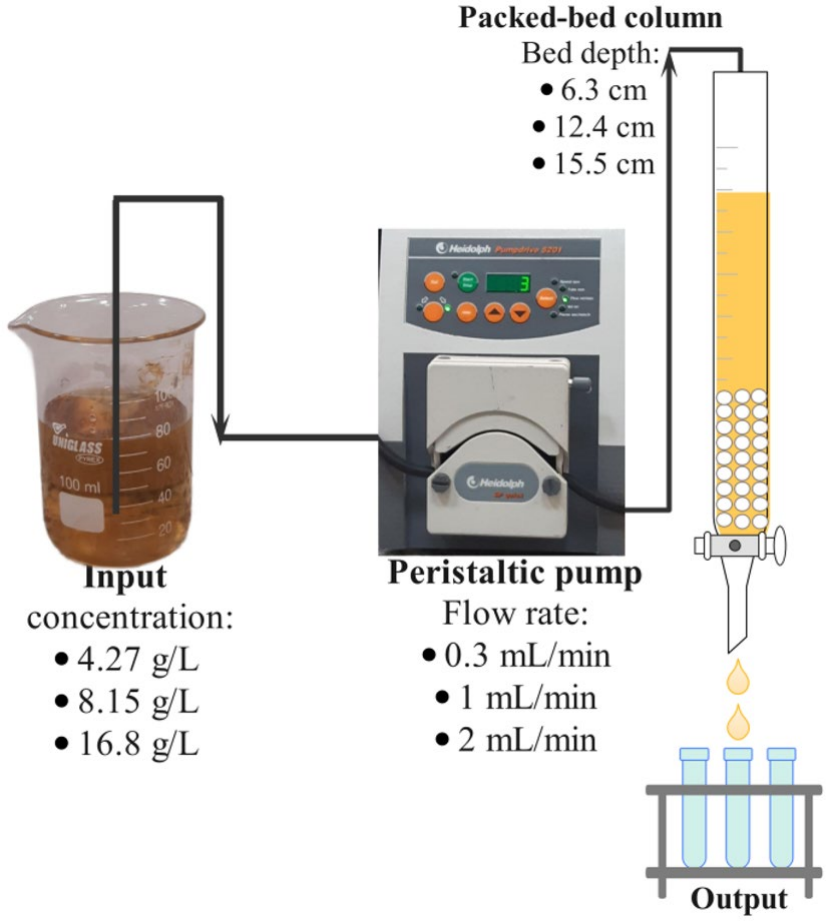
Diagram of continues fixed bed column process.

A Pyrex glass tube column with a diameter of 10 mm and a height of 31 cm was used to study fixed-bed adsorption processes. The column was packed with DAX-8 resins, and the bottom was covered with glass wool to prevent the adsorbent from escaping (Fig. 1). The DAX 8 resin, obtained from Sigma-Aldrich, is classified as a hydrophilic acrylic ester. It exhibits a relatively low ion exchange capacity of 10^−2^ Mequiv/g, possesses an average pore size of 225 Å, and demonstrates a surface area of 160 m^2^/g^33^. A peristaltic pump maintained a constant flow rate of HS solution of a specified concentration through the column. The bed heights were 6.3, 12.4, and 15.5 cm (equivalent to 2.07 g, 2.96 g, and 3.90 g of DAX-8 resins, respectively). Input HS concentrations were 4.27, 8.15, and 16.8 g/L. Also, the flow rates employed were 0.3, 1, and 2 mL/min. At periodic intervals, samples were collected to determine the remaining total organic carbon (TOC) concentration. The collection of samples continued until the concentrations of HS in the input and output leachate samples were equal.

#### Analyzing column data

The performance of fixed-bed adsorption columns was evaluated by analyzing the BTC_s_. The BTC_s_ were generally represented by the ratio C_t_/C_0_, where C_0_ is the HS input concentration (mg/L), and C_t_ is the HS concentration at time t, respectively. The time at which output HS concentrations of MSWL samples reached 5% of the input HS concentration is defined as break-through time (t_b_). Exhaustion time (t_e_) occurs when output HS concentrations exceed 95% of input concentrations^34,35^. The experimental design parameters were determined for the BTC_s_ of the column for FA adsorption onto DAX-8 resin. In this type of analysis, the top part of the BTC_s_ represents the overall adsorbate weight (q_total_, mg)^36^, which can be calculated using Eq. (1):

1 $${\text{q}}_{\text{total }}=\frac{{\text{Q}}{\text{C}}_{0}}{1000}{\int }_{0}^{{\text{t}}_{\text{t}}} \left(\text{1} - \frac{{\text{C}}_{\text{t}}}{{\text{C}}_{0}}\right)$$

where, Q represents the flow rate of HS (mL/min), C_0_ is the HS input concentration (mg/L), C_t_ is the HS concentration at time t, and t is the time (min).

Using the following equation, the volume of output, V_out_ (mL), was computed as:

2 $$ {\text{V}}_{{{\text{out}}}} = {\text{Q}} \times {\text{t}}_{{\text{e}}} $$

The equilibrium capacity to adsorption FA (q_e_, mg/g) can be calculated using Eq. (3):

3 $${\text{q}}_{\text{e}}=\frac{{\text{q}}_{\text{total }}}{\text{M}}$$

where, M (g) is the mass of the DAX-8 resin.

Finally, total FA extraction efficiency (%) can be determined as follows^37^:

4 $$ {\text{R}}\% = \frac{{1000{\text{q}}_{{{\text{total}}}} }}{{{\text{C}}_{0} {\text{Qt}}_{{\text{e}}} }} \times 100\% $$

#### Break-through curve modeling

The fixed-bed column model might predict BTC_s_ accurately in several different situations. These predictions allow us to identify operational conditions without conducting additional full-scale tests^26^. In this study, the BTC_s_ were fitted using the Thomas, Yoon-Nelson, and BDST models.

#### Thomas model

The Thomas model is used to make predictions about the adsorption capacity. Also, it evaluates the BTC_s_ performance by estimating the correlation between output concentration (C_t_) and time (t)^38^. The nonlinear Thomas model is stated as follows^39^:

5 $$\frac{{\text{C}}_{\text{t}}}{{\text{C}}_{0}}=\frac{1}{\text{1+exp }\left(\frac{{\text{k}}_{\text{T}}{{\text{q}}}_{\text{T}}{\text{m}}}{\text{Q}}-{\text{k}}_{\text{T}}{{\text{C}}}_{0}{\text{t}}\right)}$$

where, C_0_ is the HS input concentration (mg/L), C_t_ is HS concentration at time t (mg/L), t is the time (min), K_T_ is the rate constant of Thomas (mL/mg min), Q denotes the flow rate (L/min), m is the mass of the DAX-8 resin (g), and q_T_ is the capacity of column adsorption (mg/g).

#### Yoon-Nelson model

The Yoon-Nelson model is frequently used in single adsorbate systems due to its ability to disregard adsorbate properties and adsorbent kinds^35^. The nonlinear Yoon-Nelson model can be described as:

6 $$ \frac{{{\text{C}}_{{\text{t}}} }}{{{\text{C}}_{{\text{o}}} }} = \frac{1}{{1 + \exp \left( {{\text{k}}_{{{\text{YN}}}} \tau - {\text{k}}_{{{\text{YN}}}} {\text{t}}} \right)}} $$

where, K_YN_ is the rate constant of Yoon-Nelson (L/mg min), and τ is the time required for a 50% FA break-through (min).

#### BDST model

The most common and essential form of the model in the fixed-bed process is the BDST model, made by Hutchins and based on a proposal by Bohart and Adams^22^. It shows that the adsorption parameters at any bed height may be efficiently estimated using a linear correlation with the service time (t) and the bed depth (H)^40^. The BDST model can be expressed in a linear form as:

7 $$ {\text{t}} = \frac{{{\text{N}}_{0} }}{{{\text{C}}_{0} \nu }}{\text{H}} - \frac{1}{{{\text{C}}_{0} K_{BDST} }}{\text{In}}\left( {\frac{{{\text{C}}_{0} }}{{{\text{C}}_{{\text{t}}} }} - 1} \right) $$

where, K_BDST_ is the rate constant of BDST (L/mg min), H is the bed depth (cm), N_0_ is the capacity of the column adsorption (mg/L), and the velocity of the column when it is empty, denotes by ν (cm/min).

The error between the measured and predicted adsorption parameters can be obtained as:

8 $$\varepsilon =\frac{\left|{x}_{\mathrm{exp}}-{x}_{cal}\right|}{{x}_{\mathrm{exp}}}\times \text{100\%}$$

where, x_exp_ indicates the empirical data and x_cal_ is calculated based on the adsorption model.

#### Error analysis

To determine the most suitable model based on its goodness of fit with the experimental data, it is imperative to conduct a thorough analysis of the data utilizing error analysis techniques. This study employed four distinct error analyses, namely the sum of the squares of the errors (SSE), the sum of absolute errors (SAE), average relative errors (ARE), and average relative standard error (ARS), to assess the magnitude of the error. The corresponding equations for each analysis can be found in Table S1.

#### Artificial neural network modeling

In this work, the ANN approach was utilized to assess the extraction performance of FA. The ANN model comprises different layers, including the input layer, hidden neurons, the output layer, the connection weight and biases, the activation function, and the summation nod^26,35^. The R2020a version of MATLAB software is used for implementing the ANN model. To this end, the experimental findings were divided into three categories: training (70%), validation (15%), and testing (15%). The Levenberg–Marquardt algorithm was applied to train the ANN. Also, the tan-sigmoid and linear transfer functions were used as the hidden and output layers activation functions, respectively.

The evaluation of the ANN model was conducted using performance metrics, including the Mean Absolute Error (MAE), Mean Squared Error (MSE), Root Mean Squared Error (RMSE), Coefficient of Determination (R^2^), and Index of Agreement (IA). The equations for each of the performance measures are presented in Table S2.

#### Sensitivity analysis

The weight matrix approach was used to evaluate the relative importance of flow rate, input concentration, bed height, and total output time on the FA adsorption capacity. Garson developed this approach as^41^:

9 $${\mathrm{I}}_{\mathrm{j}}=\frac{\sum_{\mathrm{m}=1}^{\mathrm{m}={\mathrm{N}}_{\mathrm{h}}} \left(\left(\frac{\left|{\mathrm{W}}_{\mathrm{jm}}^{\mathrm{ih}}\right|}{\sum_{\mathrm{k}=1}^{\mathrm{Ni}} \left|{\mathrm{W}}_{\mathrm{km}}^{\mathrm{ihh}}\right|}\right)\times \left|{\mathrm{W}}_{\mathrm{mn}}^{\mathrm{ho}}\right|\right)}{\sum_{\mathrm{k}=1}^{\mathrm{k}=\mathrm{Ni}} \left\{\sum_{\mathrm{m}=1}^{\mathrm{m}={\mathrm{N}}_{h}} \left(\frac{\left|{\mathrm{W}}_{\mathrm{km}}^{\mathrm{ih}}\right|}{\sum_{\mathrm{k}=1}^{\mathrm{Ni}} \left|{\mathrm{W}}_{\mathrm{km}}^{\mathrm{ih}}\right|}\right)\times \left|{\mathrm{W}}_{\mathrm{mn}}^{\mathrm{ho}}\right|\right\}}\times 100$$

I_j_ is the relative importance of the jth input variable. Input variables are indicated by N_i_, while N_h_ indicates hidden layer neurons. Connection weight is represented by W; input, hidden, and output layers are represented by the letters *i*, h, and o. Also, a number of input, hidden, and output neurons are represented by the letters k, m, and n.

## Results and discussion

### FTIR spectroscopy

**Figure 2:**
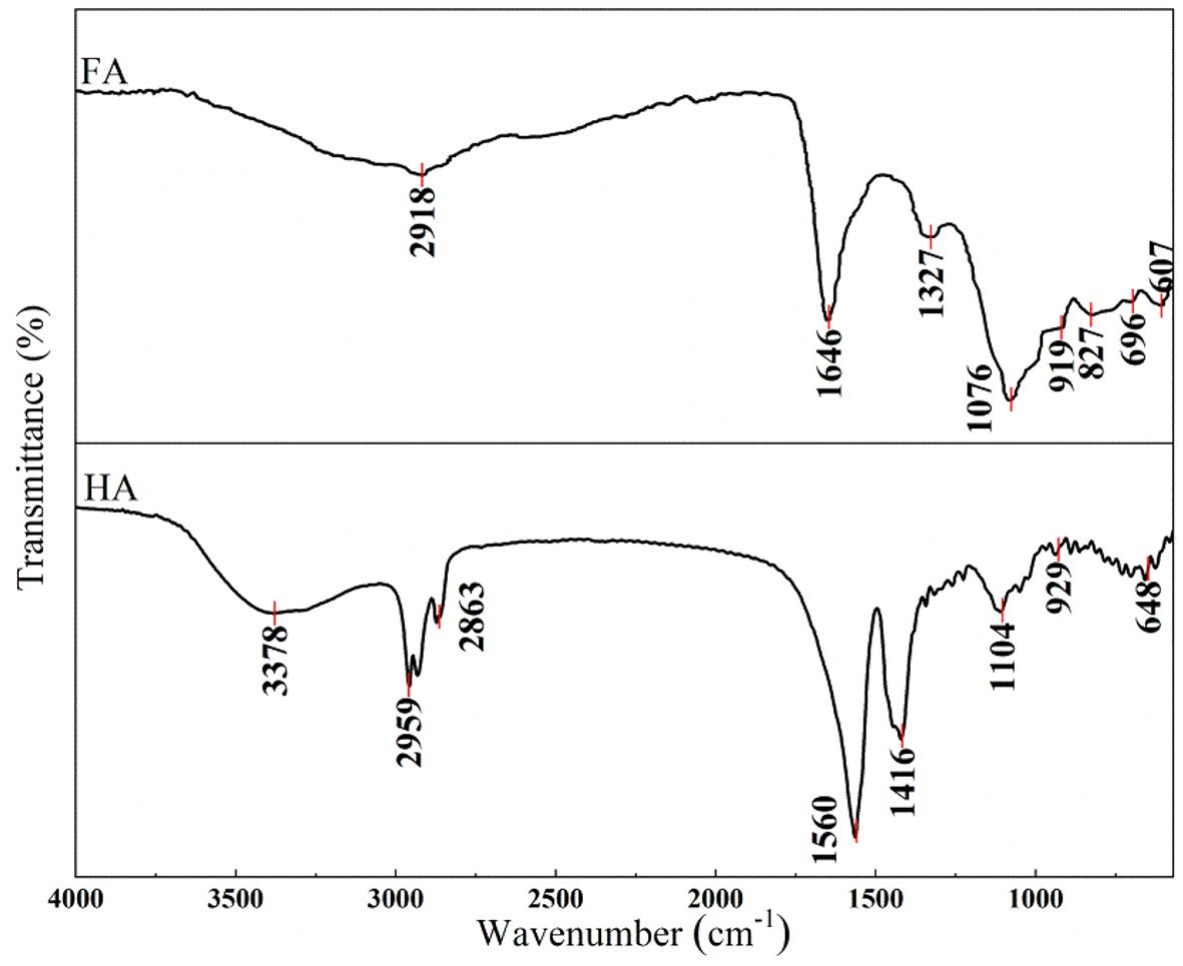
FTIR spectra of HA and FA from MSWL.

The FTIR spectra for HA and FA extracted from MSWL are shown in Fig. 2. The spectra of the basic chemical structures of HA and FA revealed some identical and some different bands. The peak of 3378 cm^−1^ is found in the spectrum of HA, related to of phenolic and alcoholic O–H group of humic acid^42^. The HA and FA spectra contain a prominent peak at 2959–2863 cm^−1^ and are assigned to aliphatic C–H^43^. The high peak at 1560 cm^−1^ in the HA spectrum is attributed to C–O of carboxylic groups^44^. The band at about 1646 cm^−1^ in the FA spectrum may be ascribed to the presence of amide groups, quinones, and ketones^45^. The peak at 1416 and 1327 cm^−1^ in HA and FA is related to C–H, C–O stretching of polysaccharides^46^. The bands around 1104 and 1076 cm^−1^ were related to aromatic C–C (in ring) groups^47^. HA and FA exhibited peaks in the 929–607 cm^−1^ range are related to mineral components and aromatic C-H (out of plane) vibrations, respectively^48^.

### Influence of flow rate on FA extraction

**Figure 3:**
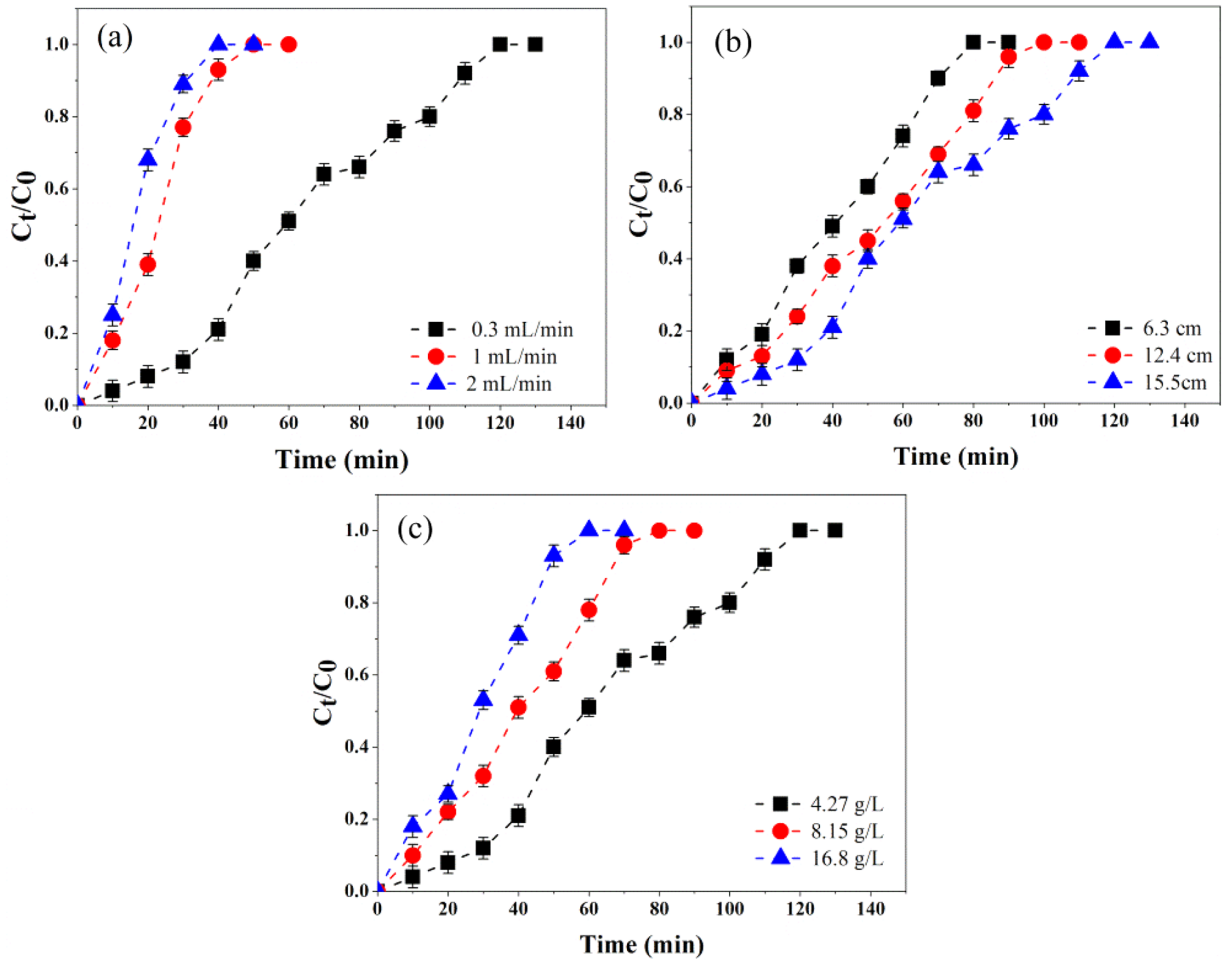
Effects of flow rate (**a**), bed height (**b**), and input concentration (**c**) on BTC_s_ of FA adsorption.

The effect of flow rate on FA extraction was studied using input flow rates of 0.3, 1, and 2 mL/min at an input concentration of 4.27 g/L and a bed height of 15.5 cm. Figure 3a depicts the BTC_s_ of FA at three flow rates, and Table 2 shows the analysis of break-through parameters for the continuous adsorption of FA. Increasing the flow rate decreased column break-through time (t_b_). At flow rates of 0.3 mL/min and 2 mL/min, the t_b_ was 12.5 min and 2.77 min, respectively. In addition, a higher adsorption capacity was observed at lower flow rates (23.03, 20.47, and 19.27 mg/g for applied flow rates of 0.3, 1, and 2 mL/min, respectively). When the flow rate is low enough, the functional groups on the resin have adequate time to engage with the FA. The output concentration quickly reaches the initial value when the system flow rate increases, resulting in an early break-through^26,49^.

**Table 2:** The break-through parameters for FA adsorption at different operating conditions.

Q (mL/min)	C_0_ (mg/L)	H (cm)	t_b_ (min)	t_e_ (min)	V_eff_ (mL)	q_total_ (mg)	q_e_ (mg/g)	R (%)
0.3	4270	15.5	12.5	113.75	34.13	81.47	23.03	61.63
1.0	4270	15.5	3.84	64.44	64.44	149.87	20.47	29.02
2.0	4270	15.5	2.77	54.99	109.98	233.99	19.27	16.07
0.3	4270	15.5	12.5	113.75	34.12	81.471	23.03	61.63
0.3	8150	15.5	5	77.85	23.35	102.69	23.82	48.82
0.3	16,800	15.5	2.77	62.85	18.85	157.75	24.55	30.23
0.3	4270	6.3	4.16	75.00	22.50	52.26	21.66	46.67
0.3	4270	12.4	5.5	89.33	26.80	66.48	22.94	59.33
0.3	4270	15.5	12.5	113.75	34.12	81.47	23.03	61.63

### Influence of bed height on FA extraction

The bed height of a continuous adsorption system is a critical design factor because it determines how long the solution stays in the column and how many adsorption sites are available^50^. Figure 3b shows the BTC_s_ for FA adsorption for three different bed heights of 6.3, 12.4, and 15.5 cm with a flow rate of 0.3 mL/min and an input concentration of 4.27 g/L. As indicated in Table 2, the t_b_ increased from 4.16 to 12.5 min when the height of the bed rose from 6.3 to 15.5 cm. In addition, increasing the height of the column improved the adsorption capacity because more surface active places of DAX-8 resin were available for FA (21.66, 22.94, and 23.03 mg/g for applied bed high of 6.3, 12.4, and 15.5 cm, respectively). Increasing the bed height increases the time required to reach break-through and exhaustion^51^. Therefore, more extended break-through and exhaustion times were seen for the column with the deeper bed height because more DAX-8 adsorbent could be contained within the column, providing more functional groups for binding with FA^26^. The height of the adsorbent bed has a significant impact on the capture of FA during dynamic adsorption. It is evident that a larger bed column is preferable for FA isolation; however, the time needed should be carefully evaluated^35,49^.

### Influence of the input concentration on FA extraction

Three different values were employed to examine the Influence of input concentration on BTC_s_ (4.27, 8.15, and 16.8 g/L), along with a bed height of 15.5 cm and flow rate of 0.3 mL/min. The results are shown in Table 2 and Fig. 3c. Increasing the input concentrations resulted in shorter break-through time and exhaustion times, which may have caused fast saturation of adsorption sites^52^. When the concentration increased from 4.27 to 16.8 g/L, the adsorption capacity increased from 23.03 to 24.55 mg/g (Table 2). Because the mass transfer driving power of the adsorption process increases with increasing input concentration, FA quickly saturates the adsorption sites on the resin^53^. The extraction rate was reduced from 61.63 to 30.23 with a higher input concentration. This is because as the input concentration increases, the output concentration increases. Simultaneously, a slower extraction rate results from a shorter break-through time.

### Data modeling for BTC_s_

Adsorption kinetics and break-through parameters from the experimental data should be estimated using the Thomas, Yoon Nelson, and BDST models.

### Thomas model

**Figure 4:**
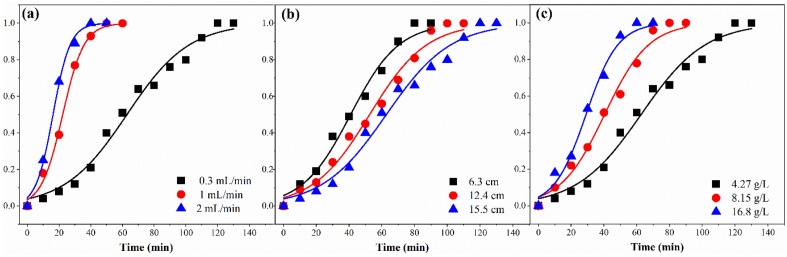
The predicted BTC_s_ for FA adsorption by the Thomas model, (**a**) flow rate; (**b**) bed height; (**c**) input concentration.

The k_T_ and q_T_ for the Thomas model were obtained by fitting Eq. (5) to experimental data. Parameters and the results of fitting the Thomas model to the experimental data are displayed in Table 3 and Fig. 4, respectively. From Table 3, the R^2^ levels (0.9857–0.9961) obtained from nonlinear fittings were relatively high, and the error levels (0.22–3.23) were low. By increasing the bed height, k_T_ valves decreased, whereas q_T_ values showed an increasing trend.

**Table 3:** Fitting the results of the dynamic adsorption model by Thomas.

Q (mL/min)	C_0_ (mg/L)	H (mL)	K_T_/(mL/mg min)	q_T_ (mg/g)	q_e, exp_	R^2^	SSE	SAE	ARE	ARS
0.3	4270	15.5	0.011	22.98	23.03	0.9858	0.295	0.543	0.136	0.039
1.0	4270	15.5	0.042	20.17	20.47	0.9961	0.018	0.136	0.047	0.019
2.0	4270	15.5	0.082	19.01	19.27	0.9954	0.015	0.121	0.019	0.009
0.3	4270	15.5	0.011	22.98	23.03	0.9858	0.295	0.543	0.136	0.039
0.3	8150	15.5	0.010	23.51	23.82	0.9880	0.117	0.341	0.056	0.019
0.3	16,800	15.5	0.009	24.15	24.55	0.9901	0.066	0.256	0.066	0.025
0.3	4270	6.3	0.019	20.96	21.66	0.9857	0.125	0.353	0.055	0.018
0.3	4270	12.4	0.012	22.61	22.94	0.9870	0.176	0.419	0.062	0.019
0.3	4270	15.5	0.011	22.98	23.03	0.9858	0.295	0.543	0.136	0.039

Conversely, by increasing the flow rate, the k_T_ values increased, but the value of q_T_ decreased. At high flow rates, FA only remains in the column for a short period, resulting in a decrease in adsorption capacity due to reduced transport rate from the surface of the adsorbent. A higher input HS concentration increased the q_0_ level but decreased the k_T_ level. This is due to increased mass transfer driving power and duration of contact between the resin and FA^35^. The adsorption capacity obtained from experimental data (q_e,exp_) is relatively close to the q_T_.

### Yoon-Nelson model

**Figure 5:**
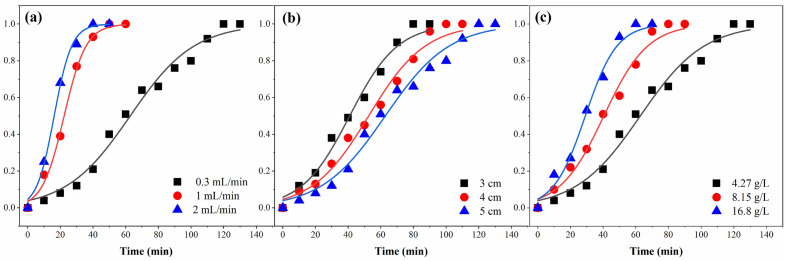
The predicted BTC_s_ for FA adsorption by the Yoon-Nelson model, (**a**) flow rate; (**b**) bed height; (**c**) input concentration.

The results of fitting the nonlinear Yoon-Nelson model to the experimental data of FA adsorption are shown in Fig. 5. The parameters K_YN_ and τ for the model are tabulated in Table 4. The values of R^2^ are above 0.98, indicating that the Yoon-Nelson model correctly describes the FA adsorption process. The value of parameter K_YN_ showed an increasing trend with increasing flow rate and input concentration. However, τ levels decreased, indicating that the contact time is insufficient for FA and the required site for resin adsorption. K_YN_ levels gradually decrease with increasing adsorbent bed height, while τ levels showed an increasing trend. By increasing the mass of DAX-8, the contact of FA with the adsorbent bed is facilitated, resulting in a prolonged column adsorption process. It is seen that the τ_cal_ levels found with the Yoon-Nelson model and τ_exp_ levels in the column adsorption study are close to each other.

**Table 4:** Fitting the results of the dynamic adsorption model by Yoon-Nelson.

Q (mL/min)	C_0_ (mg/L)	H (mL)	K_YN_/10^–3^ (mL/mg min)	τ_cal_ (min)	τ_exp_ (min)	R^2^	SSE	SAE	ARE	ARS
0.3	4270	15.5	0.051	64.02	64.47	0.9858	0.295	0.543	0.136	0.039
1.0	4270	15.5	0.092	37.77	37.09	0.9961	0.018	0.136	0.047	0.019
2.0	4270	15.5	0.095	38.95	39.21	0.9954	0.015	0.121	0.019	0.009
0.3	4270	15.5	0.051	64.02	64.47	0.9858	0.295	0.543	0.136	0.039
0.3	8150	15.5	0.062	48.39	48.74	0.9880	0.117	0.341	0.056	0.019
0.3	16,800	15.5	0.063	47.46	46.67	0.9901	0.066	0.256	0.066	0.025
0.3	4270	6.3	0.067	41.49	41.41	0.9857	0.125	0.353	0.055	0.018
0.3	4270	12.4	0.059	51.35	51.92	0.9870	0.176	0.419	0.062	0.019
0.3	4270	15.5	0.051	64.02	67.47	0.9858	0.295	0.543	0.136	0.039

### BDST model

**Figure 6:**
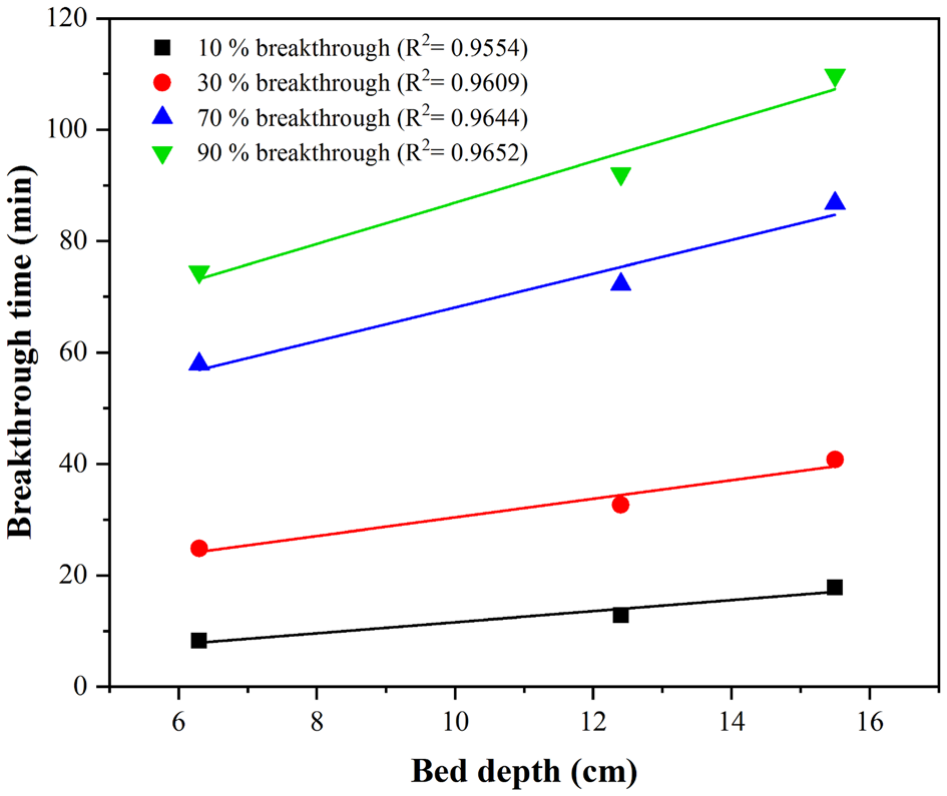
BDST model result of FA adsorption process at different break-through points.

In order to calculate the K_BDST_ and N_0_ in the BDST model, Eq. (7) used experimental data to plot the correlation between service time at the break-through point and packed-bed column depth^54^. Figure 6 shows the linear fit and model parameters of the BDST at 10%, 30%, 70%, and 90% break-through points, respectively (Table S3). The correlation coefficients of this model were obtained between. 9554 and 0.9652. By increasing the break-through point from 0.1 to 0.9, the N_0_ value increased from 77.32 mg/L to 288.06 mg/L, and the K_BDST_ value reduced from 0.086 L/mg min to 0.024 L/mg min. It shows that the diffusion rate of FA from the leachate to the adsorbent has decreased due to the reduction of the concentration gradient^55^.

### Comparison of dynamic adsorption models

In addition to the R^2^, the optimal breakthrough models for FA adsorption in column studies were determined by employing different error functions, as indicated in Table S1. This study employed four distinct error analyses to evaluate the performance of three models: Thomas, Yoon-Nelson, and BDST model. The error values, namely SSE, SAE, ARE, and ARS, obtained for Thomas and Yoon Nelson's model are presented in Tables 3 and 4. Typically, smaller error values indicate a stronger alignment between the breakthrough model and the experimental data, suggesting a better fit. The error values decreased with the increase in initial concentrations, flow rate, and bed heights. The error value calculated using the Thomas and Yoon Nelson model is also minimal compared to the BDST model. In summary, the Yoon-Nelson and Thomas models effectively depicted the experimental findings.

### ANN model

**Figure 7:**
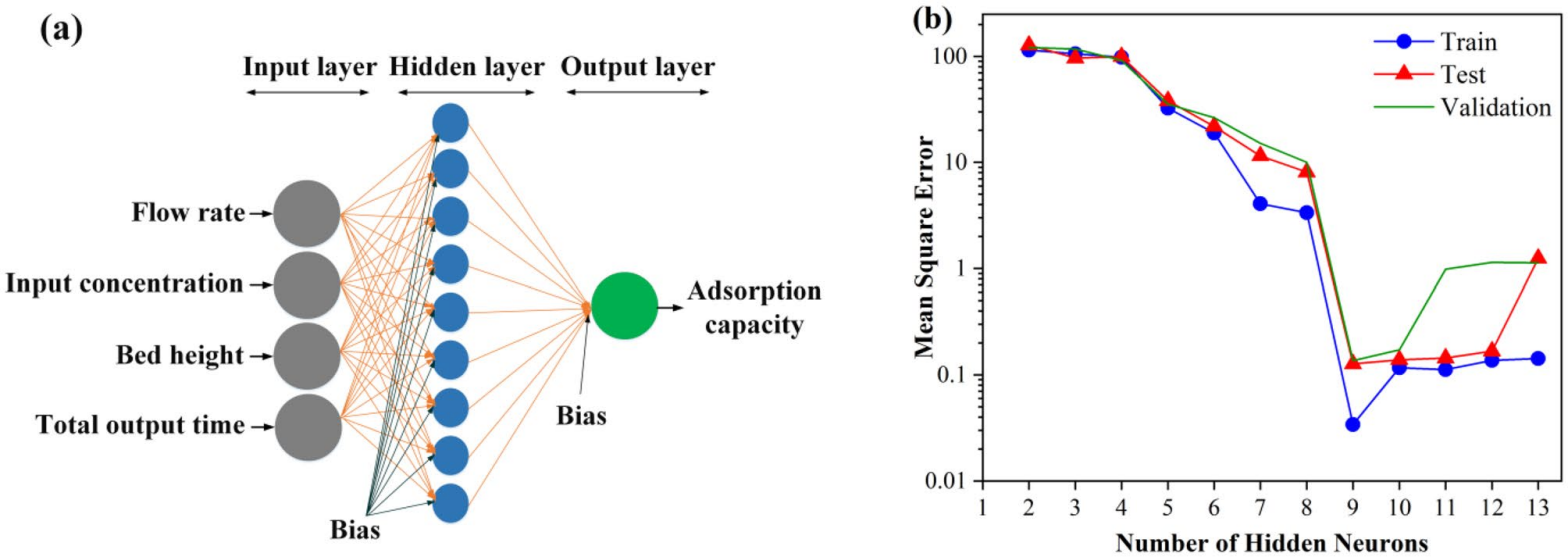
(**a**) Schematic diagram of ANN in this study; (**b**) Effect of hidden neurons.

In order to estimate the FA adsorption capacity, an ANN model with three feed-forward layers was developed, and the neural network was trained using the Levenberg–Marquardt optimization approach. Flow rate, input concentration, bed height, and total output time were used as the model inputs. On the other side, FA adsorption capacity was considered the output layer (Fig. 7a). Ranges of input and output variables and a graphical diagram of the optimized ANN are shown in Table S4 and Fig. S1, respectively. To determine the optimal number of neurons in the hidden layers, several simulations should be done with the different numbers of neurons, in which the lowest MSE is obtained^56^. The correlation between the MSE and the hidden layer neuron values is seen in Fig. 7b. The lowest MSE was obtained by nine neurons, selected as the optimized condition.

**Figure 8:**
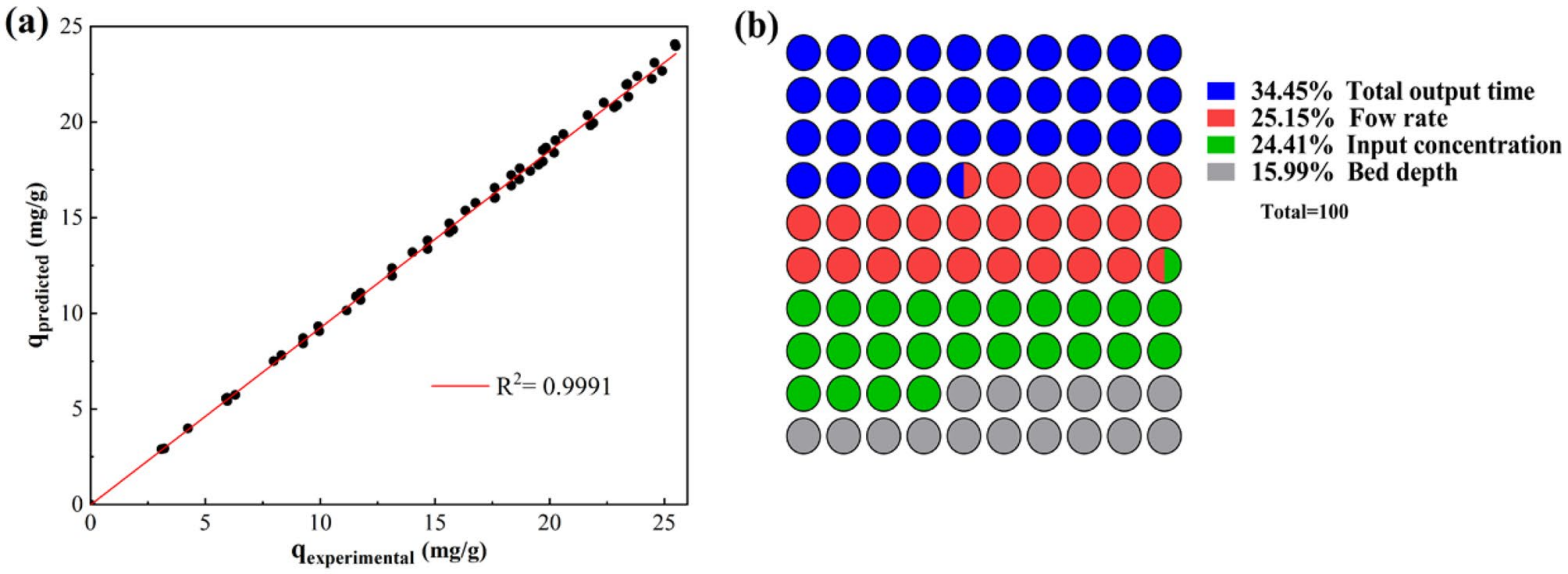
(**a**) Correlation between adsorption capacity of FA obtained from ANN model and experiments; (**b**) the relative importance of each input parameter.

The relationship between the experimental data and the predicted data by the ANN model is shown in Fig. 8a. Also, Experimental and predicted data are shown in Table S5. R^2^, MSE, RMSE, MAE, and IA parameters for this model are 0.999, 0.624, 0.790, 0.625 and 0.488, respectively. Therefore, it seems that the ANN model can accurately predict the extraction of FA in the fixed-bed column.

### Sensitivity analysis

Sensitivity analysis was utilized to estimate the level of influence of operating parameters on the Extraction of FA. Connecting the weight value of the model enlisted in Table S6 data. The importance of input parameters is evaluated by Eq. (9). The relative importance of each input variable is shown in Fig. 8b. The total output time, with a relative importance of 34.45%, was found to be the most influential variable in the adsorption of FA. After that, the relative importance of flow rate, input concentration, and column height was obtained at 25.15%, 24.41%, and 15.99%, respectively. In the process of FA adsorption from MSWL, achieving the larger adsorption capacity requires more time, so it is considered the most influential variable.

### Sorbent regeneration

**Figure 9:**
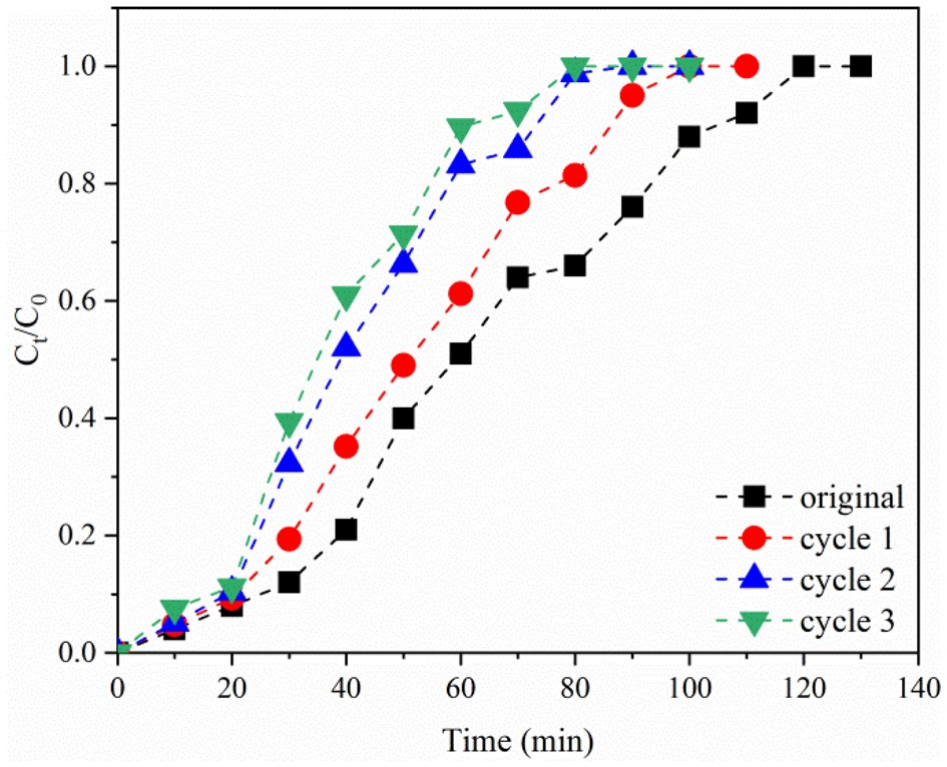
The experimental BTC_s_ for FA adsorption during regeneration cycles.

In order to extract the FA on a large-scale practically, the column must be able to be used more than once. Only in this way, the costs of the extraction process can be kept low^57^. This study evaluated the effectiveness of saturated DAX-8 resin by three adsorption–desorption cycles using 0.1 M NaOH solution. Figure 9 shows the BTC_s_ for these three regeneration cycles. The break-through time, exhaustion time, adsorption capacity, and extraction rate of each cycle are tabulated in Table S7. As can be seen, the break-through and exhaustion time in cycles 1–3 gradually decreased from 10.44 to 7.58 min and 97.21 to 83.42 min, respectively. Also, due to decreasing the break-through and exhaustion time after three adsorption–desorption cycles, the adsorption capacity and the extraction rate decreased from 17.21 to 13.11 mg/g and 49.21 to 31.47%, respectively. It should be noted that the regeneration efficiency was above 50% after three cycles. This shows that DAX-8 resin has the potential to be reused.

## Conclusions

Based on this research's objective, HA and FA extraction from MSWL was performed effectively. The extracted material was characterized by FTIR analyses, which confirmed functional groups, including carboxylic, phenolic, aliphatic, and ketone.

The influence of diversified operational variables (flow rate, input concentration, and bed height) were investigated on column performance. The break-through time, exhaustion times, adsorption capacity, and extraction rate increased with a high bed height and a low flow rate.

By increasing the input concentration, the adsorption capacity of FA increases while the break-through time, exhaustion times, and extraction rate decrease. The models proposed by Thomas and Yoon-Nelson exhibited a strong fit to the experimental column data, suggesting their ability to accurately estimate the breakthrough curves (BTC_s_). The BDST model demonstrated a strong linear correlation between bed depth and break-through point, indicating its potential suitability for column design. Based on the statistical analysis conducted, it was determined that the experimental data exhibited a strong fit with the proposed model. The ANN model had the best correlation coefficients and the smallest MSE compared to the other studied models. With relative importance of 34.45%, the total output time ranked as the most important of all the parameters. The relative importance of the flow rate, input concentration, and column height was 25.15%, 24.41%, and 15.99%, respectively. Exhausted column beds were regenerated, and adsorption capacities were changed from 41.29 to 31.47 mg/g after three cycles, indicating a relatively practical value to extract FA from MSWL.

## Supplementary Information

Supplementary Information.

## Data Availability

The datasets used and/or analyzed during the current study are available from the corresponding author on reasonable request**.**
