# Supplementary material for: Application of artificial neural network and dynamic adsorption models to predict humic substances extraction from municipal solid waste leachate
Source: Sci Rep. 2023 Aug 1;13:12421. doi: 10.1038/s41598-023-39373-2 (PMC10393967; doi:10.1038/s41598-023-39373-2)
Supplement: Supplementary file 1 — Supplementary Information. [file 41598_2023_39373_MOESM1_ESM.docx]

**Supplementary data**

**Table S1.** Equations for error analysis for fixed bed column study

| **Error functions** | **Expression** |
| --- | --- |
| Sum Absolute Errors (SAE) | SSE = ${\sum_{i=1}^{n} (b_{p}- b_{e})}_{i}^{2}$ |
| Sum Squares Errors (SSE) | SAE = $\sum_{i=1}^{n} \left\vert{(b_{p}- b_{e})}_{i} \right\vert$ |
| Average relative errors (ARE) | ARE = $\frac{1}{n}\sum_{i=1}^{n} \left\vert\frac{b_{p}- b_{e}}{b_{e}} \right\vert$ |
| average relative standard error (ARS) | ARS = $\sqrt{\sum[{\frac{\left( b_{p}- b_{e} \right)}{b_{e}}]}^{2}/n-1}$ |
| Note: $(C/{C_{o})_{mea}}$ is measured value from model equation, ${{(C}}/{C_{o})_{exp}}$is experimental value, $p$ and $n$ are number of parameters and number of data points in breakthrough model, respectively. | |

**Table S2.** The Performance Indicators measures for ANN model

| Measure | Formula |
| --- | --- |
| Coefficient of determination (R^2^) | $R^{2}=\frac{\left( \sum_{i=1}^{n_{S}} \left( d_{i}-d \right)\left( y_{i}-y \right) \right)^{2}}{\sum_{i=1}^{n_{S}} \left( d_{i}-d \right)^{2}\times\sum_{i=1}^{n_{S}} \left( y_{i}-y \right)^{2}}$ |
| mean square error (MSE) | $MSE=\frac{1}{n}\sum_{i=1}^{n} \left( y_{i}-\hat{y}_{i} \right)^{2}$ |
| Root mean square error (RMSE) | $RMSE=\sqrt{\frac{\sum_{i=1}^{N} \left( d_{i}-y_{i} \right)^{2}}{N}}$ |
| Mean absolute error (MAE) | $MAE=\frac{1}{n_{S}}\sum_{i=1}^{n_{S}} \left\vert d_{i}-y_{i} \right\vert$ |
| index of agreement (IA) | $\mathrm{IA}=1-\frac{\sum_{i=1}^{n} (d_{i}-y_{i})^{2}}{\sum_{i=1}^{n} \vert d_{i}-y_{i}\vert+\vert d_{i}-y_{i}\vert^{2}}$ |
| Note: n_s_ denote the number of observations, while d and y represent the desired (observed) and prediction output values, respectively. | |

**Table S3.** BDST model parameters at different breakthrough points

| Breakthrough point (C_t_/C_0_) | N_0_ (mg/L) | K_BDST_ (L/mg.min) | R^2^ | SSE | SAE | ARE | ARS |
| --- | --- | --- | --- | --- | --- | --- | --- |
| 0.1 | 77.32 | 0.086 | 0.9554 | 0.318 | 0.442 | 0.136 | 0.031 |
| 0.3 | 130.00 | 0.051 | 0.9609 | 0.215 | 0.331 | 0.119 | 0.022 |
| 0.7 | 235.37 | 0.014 | 0.9644 | 0.112 | 0.240 | 0.119 | 0.017 |
| 0.9 | 288.06 | 0.024 | 0.9652 | 0.011 | 0.211 | 0.092 | 0.009 |

**Table S4.** Ranges of input and output variables of ANN

| Variable | Range of the parameter value |
| --- | --- |
| **Input layer** |  |
| Input concentration (g/L) | 4.27-16.8 |
| Flow rate (mL/ min) | 0.3–2 |
| Column height (cm) | 6.3-15.5 |
| Total output time (min) | 0-110 |
| **Output layer** |  |
| Adsorption capacity (mg/g) | 0-36 |


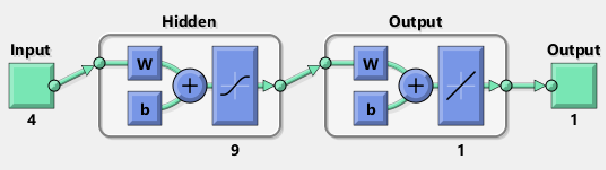


**Fig. S1.** graphical diagram of the optimized artificial neural network

**Table S5.** Experimental and predicted values of the ANN model

| Flow rate | Total effluent time | ct/c0 | Initial concentration | higth | qe_experiment_ | qe_predict_ | error (%) |
| --- | --- | --- | --- | --- | --- | --- | --- |
| 0.3 | 10 | 0.02 | 4270 | 5 | 3.22 | 3.02 | 2.28 |
| 0.3 | 20 | 0.03 | 4270 | 5 | 6.37 | 6.08 | 5.42 |
| 0.3 | 30 | 0.04 | 4270 | 5 | 9.46 | 8.11 | 8.60 |
| 0.3 | 40 | 0.05 | 4270 | 5 | 12.48 | 10.81 | 11.61 |
| 0.3 | 60 | 0.21 | 4270 | 5 | 15.57 | 14.29 | 14.65 |
| 0.3 | 70 | 0.3 | 4270 | 5 | 16.09 | 15.04 | 15.16 |
| 0.3 | 80 | 0.51 | 4270 | 5 | 12.87 | 11.87 | 11.95 |
| 0.3 | 90 | 0.67 | 4270 | 5 | 9.75 | 8.74 | 8.85 |
| 0.3 | 100 | 0.84 | 4270 | 5 | 5.25 | 4.39 | 4.41 |
| 0.3 | 110 | 0.95 | 4270 | 5 | 1.81 | 1.64 | 0.90 |
| 1 | 10 | 0.02 | 4270 | 5 | 10.73 | 10.01 | 9.80 |
| 1 | 20 | 0.05 | 4270 | 5 | 20.80 | 21.31 | 19.78 |
| 1 | 30 | 0.14 | 4270 | 5 | 28.24 | 28.02 | 27.25 |
| 1 | 40 | 0.36 | 4270 | 5 | 28.02 | 27.78 | 27.03 |
| 1 | 50 | 0.61 | 4270 | 5 | 21.35 | 22.36 | 20.30 |
| 1 | 60 | 0.76 | 4270 | 5 | 15.76 | 14.05 | 14.87 |
| 1 | 70 | 0.95 | 4270 | 5 | 3.83 | 4.02 | 2.78 |
| 2 | 10 | 0.05 | 4270 | 5 | 20.80 | 20.81 | 19.80 |
| 2 | 20 | 0.15 | 4270 | 5 | 37.22 | 37.22 | 36.22 |
| 2 | 30 | 0.29 | 4270 | 5 | 46.64 | 46.49 | 45.64 |
| 2 | 40 | 0.52 | 4270 | 5 | 42.04 | 41.48 | 41.05 |
| 2 | 50 | 0.79 | 4270 | 5 | 22.99 | 23.06 | 21.99 |
| 2 | 60 | 0.95 | 4270 | 5 | 6.57 | 6.51 | 5.58 |
| 0.3 | 10 | 0.05 | 4270 | 5 | 3.12 | 4.01 | 1.83 |
| 0.3 | 20 | 0.1 | 4270 | 5 | 5.91 | 6.08 | 4.88 |
| 0.3 | 30 | 0.27 | 4270 | 5 | 7.19 | 7.11 | 6.20 |
| 0.3 | 40 | 0.51 | 4270 | 5 | 6.44 | 7.01 | 5.35 |
| 0.3 | 50 | 0.95 | 4270 | 5 | 0.82 | 0.70 | -0.03 |
| 0.3 | 10 | 0.04 | 4270 | 5 | 3.15 | 4.61 | 1.69 |
| 0.3 | 20 | 0.05 | 4270 | 5 | 6.24 | 7.24 | 5.08 |
| 0.3 | 30 | 0.12 | 4270 | 5 | 8.67 | 9.27 | 7.60 |
| 0.3 | 40 | 0.32 | 4270 | 5 | 8.93 | 10.73 | 7.73 |
| 0.3 | 50 | 0.49 | 4270 | 5 | 8.37 | 9.21 | 7.27 |
| 0.3 | 60 | 0.68 | 4270 | 5 | 6.30 | 7.26 | 5.15 |
| 0.3 | 70 | 0.89 | 4270 | 5 | 2.53 | 3.05 | 1.32 |
| 0.3 | 80 | 0.95 | 4270 | 5 | 1.31 | 1.37 | 0.26 |
| 0.3 | 10 | 0.01 | 4270 | 5 | 3.25 | 4.70 | 1.80 |
| 0.3 | 20 | 0.03 | 4270 | 5 | 6.37 | 7.04 | 5.26 |
| 0.3 | 30 | 0.05 | 4270 | 5 | 9.36 | 10.94 | 8.19 |
| 0.3 | 40 | 0.12 | 4270 | 5 | 11.56 | 11.57 | 10.56 |
| 0.3 | 50 | 0.21 | 4270 | 5 | 12.97 | 12.85 | 11.98 |
| 0.3 | 60 | 0.3 | 4270 | 5 | 13.97 | 14.26 | 12.95 |
| 0.3 | 70 | 0.51 | 4270 | 5 | 11.26 | 11.42 | 10.25 |
| 0.3 | 80 | 0.67 | 4270 | 5 | 8.67 | 9.15 | 7.61 |
| 0.3 | 90 | 0.84 | 4270 | 5 | 4.72 | 5.44 | 3.57 |
| 0.3 | 100 | 0.95 | 4270 | 5 | 1.64 | 2.39 | 0.18 |
| 0.3 | 10 | 0.05 | 4270 | 3 | 5.87 | 5.81 | 4.88 |
| 0.3 | 20 | 0.09 | 4270 | 3 | 11.26 | 11.46 | 10.24 |
| 0.3 | 30 | 0.27 | 4270 | 3 | 13.55 | 13.41 | 12.56 |
| 0.3 | 40 | 0.51 | 4270 | 3 | 12.13 | 12.07 | 11.13 |
| 0.3 | 50 | 0.77 | 4270 | 3 | 7.11 | 7.26 | 6.09 |
| 0.3 | 60 | 0.94 | 4270 | 3 | 2.23 | 2.09 | 1.29 |
| 0.3 | 10 | 0.03 | 4270 | 4 | 4.19 | 5.26 | 2.93 |
| 0.3 | 30 | 0.15 | 4270 | 4 | 11.03 | 11.04 | 10.03 |
| 0.3 | 40 | 0.32 | 4270 | 4 | 11.77 | 10.38 | 10.89 |
| 0.3 | 50 | 0.57 | 4270 | 4 | 9.30 | 9.27 | 8.30 |
| 0.3 | 60 | 0.71 | 4270 | 4 | 7.53 | 7.61 | 6.52 |
| 0.3 | 70 | 0.84 | 4270 | 4 | 4.84 | 4.77 | 3.85 |
| 0.3 | 80 | 0.96 | 4270 | 4 | 1.38 | 1.27 | 0.46 |
| 0.3 | 10 | 0.01 | 4270 | 5 | 3.25 | 4.01 | 2.02 |
| 0.3 | 20 | 0.03 | 4270 | 5 | 6.37 | 6.07 | 5.42 |
| 0.3 | 30 | 0.05 | 4270 | 5 | 9.36 | 8.11 | 8.49 |
| 0.3 | 40 | 0.12 | 4270 | 5 | 11.56 | 10.81 | 10.62 |
| 0.3 | 60 | 0.3 | 4270 | 5 | 13.79 | 12.55 | 12.88 |
| 0.3 | 70 | 0.51 | 4270 | 5 | 11.26 | 10.11 | 10.36 |
| 0.3 | 80 | 0.67 | 4270 | 5 | 8.67 | 9.53 | 7.57 |
| 0.3 | 90 | 0.84 | 4270 | 5 | 4.72 | 5.74 | 3.50 |
| 0.3 | 100 | 0.95 | 4270 | 5 | 1.64 | 1.38 | 0.80 |

**Table S6.** Weight matrix for the relative importance variables: the weights values between input and hidden layers (W_1_) and weights values between hidden and output layers (W_2_)

| Neuron | W_1_ | | | | Bias | W_2_ |
| --- | --- | --- | --- | --- | --- | --- |
|  | Input variables | | | |  | Output variables |
|  | Initial concentration | Flow rate | Column height | Total running time |  |  |
| 1 | -2.53973 | 2.922976 | 1.891169 | 2.236279 | -2.78858 | -0.86453 |
| 2 | -3.59128 | 3.256839 | 1.579198 | 2.873957 | -2.25686 | 0.75713 |
| 3 | -0.99557 | 2.934697 | -0.86836 | 3.475348 | -1.87934 | -1.91976 |
| 4 | -1.75643 | 0.695198 | 2.526052 | -3.53137 | 0.685252 | 1.126733 |
| 5 | 2.742849 | 1.939053 | 0.833603 | 0.168338 | 1.444885 | 1.433862 |
| 6 | 2.10315 | -1.94604 | 1.822615 | -3.56677 | 1.521457 | -0.97699 |
| 7 | 1.280346 | 0.629335 | 0.24298 | 3.63757 | 1.320617 | -0.44801 |
| 8 | 0.865456 | -0.64178 | -2.55656 | -1.60182 | -0.82638 | 0.188464 |
| 9 | 1.12575 | 0.856058 | -0.2989 | -2.44387 | -1.74167 | -1.70516 |

**Table S7.** The breakthrough parameters for FA acid adsorption onto DAX-8 resin for three adsorption-desorption cycles

| Cycle | t_b_ (min) | t_e_ (min) | q_eq_ (mg/g) | Extraction rate(%) | Regeneration Efficiency (100%) |
| --- | --- | --- | --- | --- | --- |
| 0 | 12.5 | 113.75 | 23.03 | 61.63 | - |
| 1 | 10.44 | 97.21 | 17.21 | 49.21 | 74.73 |
| 2 | 9.8 | 87.07 | 15.52 | 38.94 | 67.39 |
| 3 | 7.58 | 83.42 | 13.11 | 31.47 | 56.93 |

**References**

1. Baccot, C., Pallier, V., Thom, M.T., Thuret-Benoist, H., Feuillade-Cathalifaud, G., 2020. Valorization of extracted organic matter from municipal solid waste leachate: Application to soils from France and Togo. Waste Management 102, 161-169.

2. Mallick, S.P., 2017. Method Development for Aquatic Humic Substance Isolation and Its Application to Landfill Leachate. Lamar University-Beaumont.

3. Aslam, M.M.A., Den, W., Kuo, H.-W., 2021. Removal of hexavalent chromium by encapsulated chitosan-modified magnetic carbon nanotubes: fixed-bed column study and modelling. Journal of Water Process Engineering 42, 102143.

4.Bai, S., Li, J., Ding, W., Chen, S., Ya, R., 2022. Removal of boron by a modified resin in fixed bed column: Breakthrough curve analysis using dynamic adsorption models and artificial neural network model. Chemosphere 296, 134021.

5. Gupta, K.N., Kumar, R., 2021. Fixed bed utilization for the isolation of xylene vapor: Kinetics and optimization using response surface methodology and artificial neural network. Environmental Engineering Research 26.

6. Chen, S., Bai, S., Ya, R., Du, C., Ding, W., 2022. Continuous silicic acid removal in a fixed-bed column using a modified resin: Experiment investigation and artificial neural network modeling. Journal of Water Process Engineering 49, 102937.
